# Effect of transversus abdominis plane block on postoperative pain after nephrectomy: a systematic review

**DOI:** 10.3389/fmed.2026.1836287

**Published:** 2026-06-03

**Authors:** Wei Huang, JinPing Wang, Yu Du, Ji Wang

**Affiliations:** 1 Department of Anaesthesiology, Affiliated Hospital of North Sichuan Medical College, Nanchong, China; 2 Department of Anaesthesiology, The Affiliated Nanchong Central Hospital of North Sichuan Medical College, Beijing Anzhen Nanchong Hospital of Capital Medical University and Nanchong Central Hospital, Nanchong, Sichuan, China

**Keywords:** nephrectomy, postoperative analgesia, randomised controlled trial, systematic review, transversus abdominis plane block

## Abstract

**Objective:**

To systematically evaluate the effect of transversus abdominis plane block (TAPB) on postoperative analgesia after nephrectomy.

**Methods:**

A computerised search was conducted to identify randomised controlled trials (RCTs) evaluating TAPB for postoperative analgesia after nephrectomy from database inception to 31 January 2026 in the Cochrane Library, PubMed, Embase, Web of Science, China Biomedical Literature Service, Wanfang Data, and the China National Knowledge Infrastructure. Two researchers independently screened the literature, extracted data, and assessed the risk of bias of the included studies. Meta-analysis was performed using Review Manager 5.4. The quality of evidence was graded using the GRADEpro system, and publication bias for endpoint indicator was assessed using Stata 17.0.

**Results:**

Ten RCTs involving a total of 639 patients were included. The meta-analysis showed that, compared with the control group, the TAPB group demonstrated a significant reduction in intravenous morphine equivalents at 24-hour postoperative [MD = −16.67, 95% CI (−25.57, −7.77), *p* < 0.001]. Resting pain scores were significantly lower at 6-hour (*p* = 0.003), 12-hour (*p* < 0.001), and 24-hour (*p* = 0.020) postoperatively, although the difference at 2-hour (*p* = 0.160) and 4-hour (*p* = 0.100) was not statistically significant. Active pain scores were lower at 2-hour (*p* = 0.020), 4-hour (*p* < 0.001), 6-hour (*p* < 0.001), 12-hour (*p* = 0.002), and 24-hour (*p* < 0.001) postoperatively. TAPB did not prolong the time to first analgesia (*p* = 0.120) or postoperative hospital stay (*p* = 0.200), and it reduced the incidence of postoperative gastrointestinal adverse reactions (*p* < 0.001).

**Conclusion:**

Except for the absence of significant differences in resting pain scores at 2 and 4 hours postoperative, TAPB might reduce postoperative pain after nephrectomy, decreased postoperative opioid consumption, lowered postoperative pain scores, and reduced gastrointestinal adverse reactions.

## 1 Introduction

Nephrectomy is a common urological procedure, and patients frequently experience mild to moderate pain within the first 24-h after surgery, which may lead to complications such as urinary retention, constipation, and pulmonary atelectasis, thereby delaying recovery (1). Transversus abdominis plane block (TAPB) is an effective technique for reducing postoperative pain after abdominal surgery (2). It involves injecting local anaesthetics into the fascial plane between the transversus abdominis and internal oblique muscles, thereby blocking the anterior abdominal wall nerves originating from thoracic segment 6 to lumbar segment 1 and preventing nociceptive transmission to the central nervous system, producing an analgesic effect (3). TAPB has been shown to effectively reduce postoperative pain after abdominal procedures such as hysterectomy, caesarean section, rectal resection, appendectomy, transanal total mesorectal excision, and open hepatic surgery (4–9).

Clinical studies have evaluated the effect of TAPB on postoperative analgesia after nephrectomy. Some studies reported that TAPB significantly reduced postoperative opioid requirements and pain severity (10). However, one study concluded that TAPB did not significantly reduce postoperative opioid consumption after nephrectomy (11, 12). In 2016, a systematic review reported that TAPB did not demonstrate any significant advantage over controls in reducing postoperative opioid dosage or pain severity in urological surgery (13). However, that evaluation was published several years ago, and the control groups included placebo, intrathecal morphine, epidural analgesia, and ilioinguinal nerve block, while the types of surgical procedures included laparoscopic nephrectomy, radical prostatectomy, and renal transplantation, all of which differ from the inclusion criteria of the present systematic review.

Therefore, this systematic review was conducted to review and analyse available randomised controlled trials (RCTs) that assessed the analgesic effect of TAPB after nephrectomy, using either no TAPB or saline placebo as the control. The cumulative intravenous morphine equivalent within the first 24-h postoperative was used as the primary outcome, with the aim of providing a reference for postoperative analgesia selection in nephrectomy.

## 2 Materials and methods

This systematic review was carried out according to the guidelines of the Preferred Reporting Items for Systematic Reviews and Meta-Analyses (PRISMA 2020) (14). The study protocol was retrospectively registered with PROSPERO (Registration number: CRD420251235245).

### 2.1 Literature search

The computerised search covered the period from the establishment of each database to 31 January 2026. The Cochrane Library, PubMed, Embase, Web of Science, China Biomedical Literature Service System, Wipu, and China Knowledge Network databases were searched to identify RCTs evaluating TAPB for postoperative analgesia after nephrectomy without language restrictions. To identify additional eligible RCTs, the references and citations of relevant studies were manually searched. Unpublished and ongoing studies were also searched in ClinicalTrials.gov (www.clinicaltrials.gov) and the World Health Organization International Clinical Trials Registry Platform (WHO ICTRP; https://apps.who.int./trialsearch/). When necessary, authors were contacted to obtain further information. A search strategy combining Medical Subject Headings (MeSH) and free-text terms was applied, and the specific search strategy is presented in Supplementary Table S1.

### 2.2 Study selection

The studies included in this systematic review were based on the PICOS principles: (1) P (patients): adult patients undergoing nephrectomy under general anesthesia; (2) I (intervention): perioperative TAPB; (3) C (control): no TAPB or saline placebo TAPB; (4) O (outcomes): the primary outcome was the cumulative intravenous morphine equivalent within the first 24-h postoperative period (expressed in mg of morphine, where 1 mg morphine equivalent = 1 mg intravenous morphine). Secondary outcomes included pain scores at rest at 2, 4, 6, 12, and 24 h after surgery, pain scores during activity (cough or movement) at 2, 4, 6, 12, and 24 h after surgery, time to first postoperative analgesia, length of postoperative hospital stay, and incidence of postoperative gastrointestinal adverse events; (5) S (study design): randomized controlled trial, blinded or unblinded. Exclusion criteria: (1) absence, incompleteness, or obvious errors in data related to postoperative analgesic efficacy; (2) combined use of other regional nerve blocks; (3) duplicate publications; (4) studies for which the full text could not be obtained after contacting the authors three or more times.

### 2.3 Literature screening and data extraction

After importing all search results into EndNote 20 and removing duplicates, titles and abstracts were screened based on the inclusion and exclusion criteria. Full texts of studies that passed the initial screening were retrieved and assessed in detail for final inclusion. Data were extracted using XLSX worksheets to record key study information, including first author, year of publication, country, anaesthesia modality, age, body mass index (BMI), sample size, nerve block modality [medication, timing and technique (single-shot or continuous catheter-based)], background analgesia regimen, interventions, and outcome indicators. When outcome indicators were presented only in graphical form, the authors were contacted to obtain raw data. If raw data could not be obtained, the GetData Graph Digitizer software (version 2.20) was used to extract the data.

Literature screening and data extraction were performed independently by two researchers and cross-checked. Disagreements were resolved through discussion, and if no consensus was reached, a third researcher was consulted. Missing data were sought by contacting the study authors via email. If the data remained unavailable, extraction from that study was abandoned.

### 2.4 Data conversion

All studies reported opioid dosages converted to intravenous morphine equivalents (15). Pain scores at rest or during activity were standardised to a 0–100 scale by multiplying the value by 10 when a numerical rating scale (NRS) or visual analogue scale (VAS) of 0–10 was used, where 0 indicates no pain and 100 indicates extreme pain (16). When the pain assessment state (rest or activity) was not specified, it was inferred based on score patterns reported in similar studies. The time to first postoperative analgesia was recorded in minutes, and postoperative hospital stay was recorded in days. Postoperative nausea and vomiting were used to reflect gastrointestinal adverse effects. Continuous variables reported as medians with interquartile ranges or whole ranges were converted to mean difference (MD) ± standard deviation (SD) (17, 18).

### 2.5 Risk of bias evaluation

Two researchers independently assessed the risk of bias of the included RCTs using the Cochrane risk of bias 2 tool (RoB2) (19). This tool evaluates five domains: (1) Randomization process; (2) Deviations from intended interventions; (3) Missing outcome data; (4) Measurement of the outcome; and (5) Selection of the reported result. Each domain contains 3–7 signaling questions. Based on responses to these questions, the RoB 2 algorithm automatically classifies the risk of bias for each domain as “low risk,” “Some concerns,” or “high risk,” and finally generates a risk-of-bias summary figure. Final judgements were made through discussion and arbitration.

### 2.6 Statistical analysis

Two researchers independently entered the extracted data into Review Manager (version 5.4) for meta-analysis and cross-checked the entries. Results were summarised in tables. MD was used to analyse continuous variables, and relative risk (RR) was used for dichotomous variables, each with a 95% confidence interval (CI). Heterogeneity across studies was assessed using the *I^2^* statistic. When *I^2^* was ≤ 50%, heterogeneity was considered low, and a fixed-effects model was applied. When *I^2^* was > 50%, sources of heterogeneity were further analysed, and after excluding major sources of heterogeneity, a random-effects model was applied. Studies with marked heterogeneity were analysed using sensitivity analysis or subgroup analysis, with subgroup factors including region, nerve block medication, nerve block timing, and risk of bias, or were described narratively. When ten or more studies were included in the meta-analysis, processed data were imported into Stata (version 17.0) for cross-checking, and publication bias was evaluated using Egger’s test. A *p*-value < 0.05 was considered statistically significant. Since many small sample studies were included in this review, repeated tests after inclusion might increase the Type I errors in systematic review. In view of this, we introduced the software Trial Sequence Analysis (version 0.9) (TSA). The analysis was conducted under a random-effects model (SJ method) with a two-sided test (*α* = 0.05, *β* = 0.8). Variance was estimated empirically, and heterogeneity was assessed based on model variance. TSA enables the calculation of an adjusted required information size (RIS), which is predefined according to clinical relevance while maintaining an overall type I error rate of 5% and a type II error rate of 20% (80% power). Trial sequential monitoring boundaries (TSMB) and RIS were subsequently derived.

### 2.7 GRADE rating of the quality of evidence

The GRADEpro system was used to evaluate the quality of evidence for the meta-analysis outcomes. Five factors that may reduce the quality of evidence were considered: (1) risk of bias; (2) inconsistency; (3) imprecision; (4) indirectness; and (5) publication bias. Judgements for each domain were based on the information reported in the included studies and the contribution of each study to the overall effect estimate, taking into account the influence of study design and quality, heterogeneity and inconsistency across studies, precision of effect estimates as reflected by confidence intervals, applicability of populations, interventions, and outcomes, and the likelihood of publication bias (20). The quality of evidence for each outcome was classified as high, moderate, low, or very low.

## 3 Results

### 3.1 Literature inclusion

**Figure 1:**
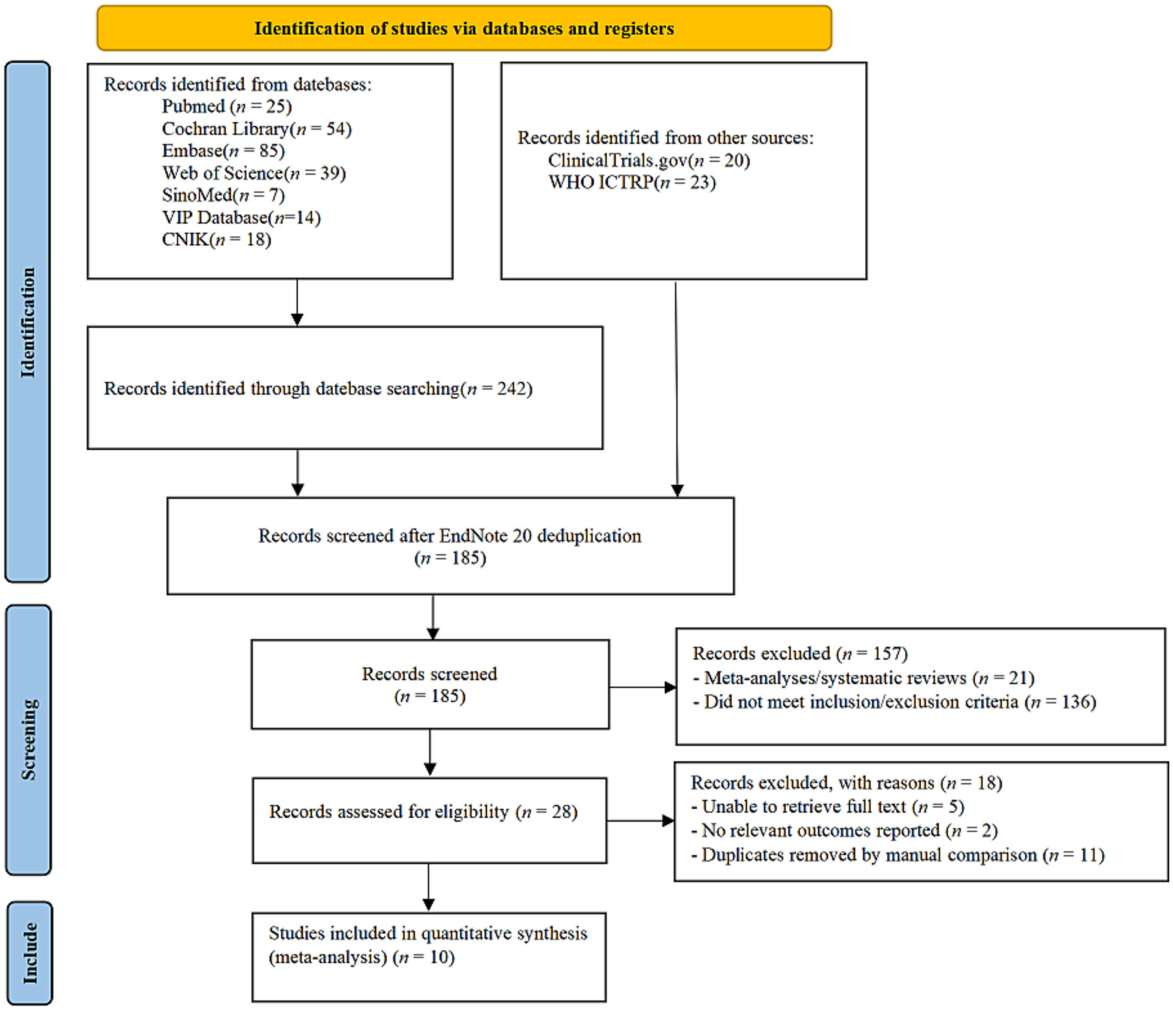
Literature screening process and results.

A total of 285 records were identified in the initial search, including 242 records from electronic databases (Cochrane Library, PubMed, Embase, Web of Science, SinoMed, VIP, and CNKI) and 43 additional records from clinical trial registries (ClinicalTrials.gov and WHO ICTRP). After duplicate removal using EndNote 20 and manual verification, 185 records remained for screening. Of these, 157 records were excluded based on title and abstract screening, including 21 meta-analyses or systematic reviews and 136 studies that did not meet the predefined inclusion criteria. The remaining 28 full-text articles were assessed for eligibility. Of these, 18 were excluded for the following reasons: 5 due to unavailable full text, 2 for not reporting relevant outcomes, and 11 identified as duplicate publications upon manual review. Ultimately, 10 randomized controlled trials (RCTs) (21–30) involving 639 patients were included in the meta-analysis, with 321 patients in the TAPB group and 318 in the control group. The literature screening process is shown in Figure 1.

### 3.2 Basic characteristics of included studies and risk of bias assessment

The basic characteristics of the included studies are presented in Table 1. Among the included studies, 2 (22, 27) were assessed as having a low risk of bias and 8 (21, 23–26, 28–30) as having some concerns of bias (Table 2).

**Table 1:** Basic characteristics of the included study.

Included study	Sample size	Country	Anesthesia Method	Study group (sample size)	Age (years) mean(SD)	BMI (Kg/m^2^) mean (SD)	Sex, No. of males (%)	TAPB technique	Intervention	Block timing	Background analgesia	Outcome measures
Aniskevich et al. (21)	21	USA	General	TAPB (10)	51.1 (16.5)	24.8 (2.7)	6 (60%)	Single-shot	0.5% Ropivacaine 20 mL	After induction (of general anesthesia)	PCIA: Morphine 1 mg bolus, 8-min lockout; increased to 1.5 mg per 8 min if analgesia inadequate	①②④⑤⑥⑦⑨⑩⑪⑫⑬⑭
				Control (11)	55.6 (12.2)	25.4 (3.7)	6 (55%)		Equivalent Saline			
Covotta et al. (22)	96	Italy	General	TAPB (48)	54.3 (14)	26.9 (5.3)	30 (62.5%)	Single-shot	0.5% Ropivacaine 30 mL	After induction (of general anesthesia)	PCIA: Morphine 0.02 mg/kg bolus; IV Acetaminophen 1 g postop, then 1 g q6h for 5 days	①③⑥⑧⑪⑬⑭
				Control (48)	60.2 (9.1)	27.7 (5.4)	22 (45.8%)		—			
Güner et al. (23)	47	Turkey	General	TAPB (25)	51.5 (10.9)	28.6 (5.3)	12 (48%)	Single-shot	0.5% Bupivacaine 20 mL	End of surgery, before extubation	PCIA: Morphine 0.05 mg/kg bolus, 10-min lockout; IV Acetaminophen 1 g postop, then 1 g q6h	①④⑤⑥⑭
				Control (22)	46.6 (11.5)	29.5 (5.2)	10 (45.5%)		Equivalent Saline			
Han and Zhou(24)	60	China	General	TAPB (30)	58 (12)	23 (3)	21 (70%)	Single-shot	0.375% Ropivacaine 20 mL	After induction (of general anesthesia)	PCIA: Fentanyl 10 μg/kg + Flurbiprofen 300 mg in 100 mL NS, 4 mL bolus, 5-min lockout, max 12 mL/h	②④⑤⑥⑦⑨⑩⑪⑭
				Control (30)	60 (11)	24 (3)	23 (76.7%)		Equivalent Saline			
Hong and Qiu(25)	72	China	General	TAPB (36)	54.12 (10.33)	22.23 (1.11)	20 (55.6%)	Single-shot	0.375% Ropivacaine 20 mL	After induction (of general anesthesia)	—	④⑤⑥⑬⑭
				Control (36)	55.01 (10.12)	22.45 (1.01)	18 (50%)		—			
Hou et al. (26)	60	China	General	TAPB (30)	47 (8)	—	18 (60%)	Single-shot	0.5% Ropivacaine 20 mL	End of surgery, before extubation	PCIA: Sufentanil 100 μg + Ondansetron 16 mg in 100 mL NS, 2 mL/h background, 1.5 mL bolus, 15-min lockout; IV Flurbiprofen 50 mg if VAS > 4	①②③④⑤⑥⑦⑧⑨⑩⑪⑬⑭
				Control (30)	48 (9)	—	17 (56.7)		Equivalent Saline			
Li et al. (27)	103	China	General	TAPB (52)	51.9 (10.3)	24.8 (3.8)	31 (60.8%)	Single-shot	0.4% Ropivacaine 30 mL	After induction (of general anesthesia)	PCIA: Sufentanil 1.25 μg/mL, background infusion 0.5 mL/h, 4 mL bolus, 10-min lockout	①③⑤⑥⑦⑨⑩⑫⑬⑭⑮⑯
				Control (51)	51.1 (11.1)	24.8 (3.3)	32 (61.5%)		Equivalent Saline			
Parikh et al. (28)	60	India	General	TAPB (30)	45 (9.6)	—	8 (26.7%)	Single-shot	0.375% Bupivacaine 25 mL	End of surgery, before extubation	IV Tramadol on demand	①②③④⑤⑥⑦⑧⑨⑩⑪⑫
				Control (30)	42 (7)	—	7 (23.3%)		Equivalent Saline			
Wang and Wen (29)	60	China	General	TAPB (30)	70.7 (2.1)	—	—	Single-shot	0.25% Ropivacaine 20 mL	After induction (of general anesthesia)	PCIA: Oxycodone 50 mg in 100 mL NS, 0 mL/h background, 4 mL bolus, 5-min lockout	④⑤⑥⑨⑩⑪⑭
				Control (30)	71.3 (2.5)	—	—		—			
Yang and Jiang (30)	60	China	General	TAPB (30)	—	—	—	Single-shot	0.33% Ropivacaine 15 mL	After induction (of general anesthesia)	PCIA: Sufentanil 1 μg/mL in 200 mL + Ondansetron 4 mg, 2 mL/h background, 0.5 mL bolus, 15-min lockout	①②③④⑤⑥⑭
				Control (30)	—	—	—		Equivalent Saline			

T, TAPB group; C, Control group; PCIA, Patient-controlled intravenous analgesia; —, Not Involving; ①, Intravenous morphine equivalent at 24-h postoperative; ②, Postoperative 2-h resting pain score; ③, Postoperative 4-h resting pain score; ④, Postoperative 6-h resting pain score; ⑤, Postoperative 12-h resting pain score; ⑥, Postoperative 24-h resting pain score; ⑦, Postoperative 2-h active pain score; ⑧, Postoperative 4-h active pain score; ⑨, Postoperative 6-h active pain score; ⑩, Postoperative 12-h active pain score; ⑪, Postoperative 24-h active pain score; ⑫, Time to first postoperative analgesia; ⑬, Duration of postoperative hospitalisation; ⑭, Incidence of postoperative gastrointestinal adverse reactions.

**Table 2:** Risk-of-bias assessment of included studies.

Included Study	Randomization process	Deviations from intended interventions	Missing outcome data	Measurement of the outcome	Selection of the reported result	Overall
Aniskevich et al. (21)	Low risk	Low risk	Low risk	Low risk	Some concerns	Some concerns
Covotta et al. (22)	Low risk	Low risk	Low risk	Low risk	Low risk	Low risk
Güner et al. (23)	Low risk	Low risk	Low risk	Low risk	Some concerns	Some concerns
Han and Zhou (24)	Some concerns	Some concerns	Low risk	Low risk	Some concerns	Some concerns
Hong and Qiu (25)	Low risk	Some concerns	Low risk	Low risk	Some concerns	Some concerns
Hou et al. (26)	Low risk	Some concerns	Low risk	Low risk	Some concerns	Some concerns
Li et al. (27)	Low risk	Low risk	Low risk	Low risk	Low risk	Low risk
Parikh et al. (28)	Low risk	Low risk	Low risk	Low risk	Some concerns	Some concerns
Wang and Wen (29)	Some concerns	Low risk	Low risk	Low risk	Some concerns	Some concerns
Yang and Jiang 2012 (30)	Low risk	Low risk	Low risk	Low risk	Some concerns	Some concerns

### 3.3 Meta-analysis results

#### 3.3.1 Intravenous morphine equivalents at 24-h postoperative

**Figure 2:**
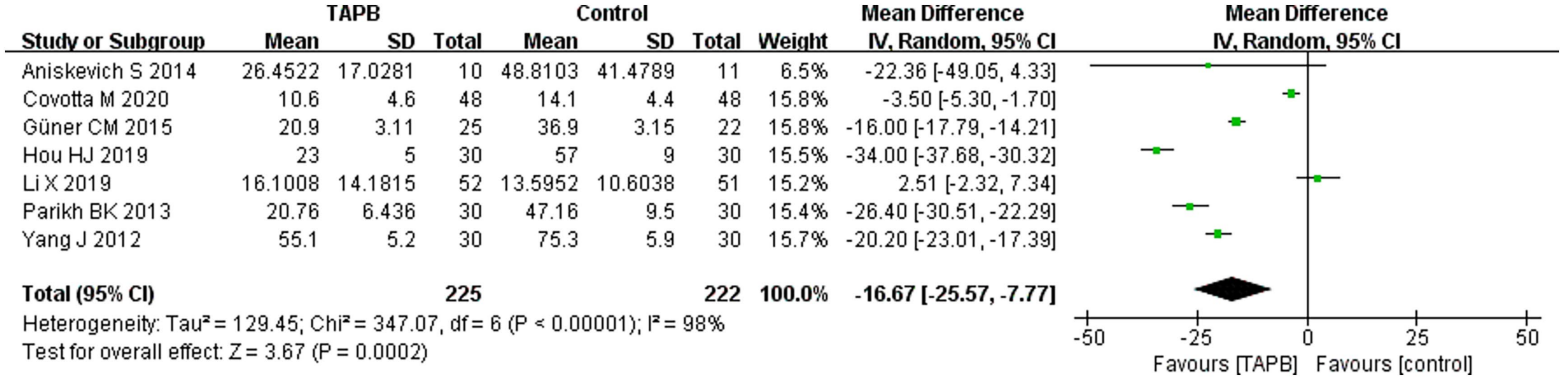
Meta-analysis of 24-h postoperative intravenous morphine equivalents.

Seven studies (21–23, 26–28, 30) compared intravenous morphine equivalents at 24-h postoperative, involving a total of 447 patients, of whom 225 were in the TAPB group and 222 were in the control group. A random-effects model was applied. The results indicated that the TAPB group required significantly fewer intravenous morphine equivalents within 24-h after surgery than the control group [MD = −16.67, 95% CI (−25.57, −7.77), *p* < 0.001]. The minimum clinically important difference (MCID) for this outcome is 10 mg (31). The observed reduction exceeds the MCID, indicating that TAPB provides a clinically meaningful reduction in early postoperative opioid use. Heterogeneity among studies was substantial (*I^2^* = 98%). Sensitivity and subgroup analyses were conducted to explore potential sources of heterogeneity; however, no individual study or subgroup was identified as having a decisive influence. Sequential exclusion of individual studies did not alter the direction of the effect, indicating that the results were stable. Details are presented in Figure 2, Table 3, and Supplementary Table S2.

**Table 3:** Meta-analysis results and evidence levels.

Outcome measure	Number of studies	Sample size	Heterogeneity		Effect model	Meta-analysis result		Quality of evidence
			*I^2^* (%)	*p*-value		Effect size (95% CI)	*p*-value	
Intravenous morphine equivalent at 24-h postoperatively	7 [21–23,26–28,30]	447	98	<0.001	Random	−16.67^a^ (−25.57, −7.77)	<0.001	⊕
Postoperative 2-h resting pain score	6 [21,24,26–28,30]	364	99	<0.001	Random	−10.24^a^ (−24.49, 4.02)	0.160	⊕⊕
Postoperative 4-h resting pain score	4 [22,26,28,30]	276	99	<0.001	Random	−14.39^a^ (−31.71, 2.93)	0.100	⊕
Postoperative 6-h resting pain score	9 [21,23–30]	543	97	<0.001	Random	−11.09^a^ (−18.35, −3.84)	0.003	⊕⊕
Postoperative 12-h resting pain score	9 [21,23–30]	543	95	<0.001	Random	−8.82^a^ (−13.81, −3.82)	<0.001	⊕
Postoperative 24-h resting pain score	10 [21–30]	639	97	<0.001	Random	−6.9^a^ (−12.83, −0.97)	0.020	⊕ ⊕ ⊕
Postoperative 2-h active pain score	5 [21,24,26–28]	304	87	<0.001	Random	−8.87^a^ (−16.36, −1.38)	0.020	⊕ ⊕ ⊕
Postoperative 4-h active pain score	3 [22,26,28]	216	0	0.380	Fixed	−11.26^a^ (−14.10, −8.42)	<0.001	⊕ ⊕ ⊕
Postoperative 6-h active pain score	6 [21,24,26–29]	364	25	0.250	Fixed	−9.51^a^ (−11.12, −7.90)	<0.001	⊕⊕
Postoperative 12-h active pain score	6 [21,24,26–29]	364	80	<0.001	Random	−8.52^a^ (−13.87, −3.18)	0.002	⊕ ⊕ ⊕
Postoperative 24-h active pain score	7 [21,22,24,26–29]	460	61	0.020	Random	−5.57^a^ (−8.84, −2.31)	<0.001	⊕ ⊕ ⊕⊕
Time to first postoperative analgesia	3 [21,27,28]	184	98	<0.001	Random	246.04^a^ (−62.04, 554.13)	0.120	⊕
Duration of postoperative hospitalisation	6 [21–23,25–27]	399	86	<0.001	Random	−0.47^a^ (−1.19, 0.25)	0.200	⊕
Incidence of postoperative gastrointestinal adverse reactions	8 [21,22,24–27,29,30]	532	0	0.470	Fixed	0.57^b^ (0.44, 0.74)	<0.001	⊕ ⊕ ⊕

a, MD: b, RR; ⊕, Very low quality of evidence; ⊕ ⊕, Low quality of evidence; ⊕ ⊕⊕, Medium evidence quality; ⊕ ⊕ ⊕⊕, High quality of evidence.

#### 3.3.2 Postoperative 2-h resting pain score

**Figure 3:**
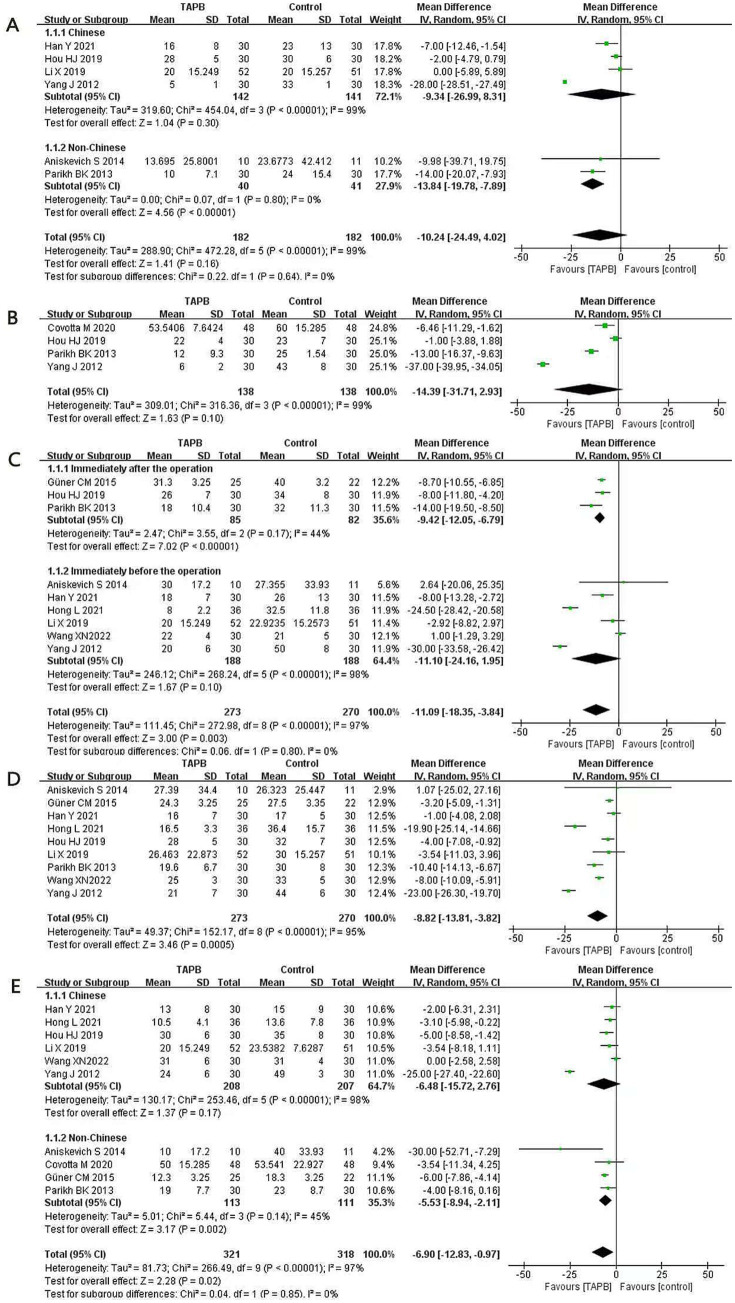
Meta-analysis of postoperative resting pain scores. **(A)** Postoperative 2-h resting pain score; **(B)** Postoperative 4-h resting pain score; **(C)** Postoperative 6-h resting pain score; **(D)** Postoperative 12-h resting pain score; **(E)** Postoperative 24-h resting pain score.

Six studies (21, 24, 26–28, 30) compared postoperative 2-h resting pain scores, including a total of 364 patients (182 in the TAPB group and 182 in the control group). A random-effects model was used. Overall, no statistically significant difference was observed between the two groups [MD = −10.24, 95% CI (−24.49, 4.02), *p* = 0.160], while differences were identified in the subgroup analysis. Due to the high heterogeneity among studies (I^2^ = 99%), sensitivity and subgroup analyses were conducted. Subgroup analyses indicated that regional differences were an important source of heterogeneity. Within the non-Chinese subgroup (21, 28), heterogeneity was low (*I^2^* = 0%), and the TAPB group (40 patients) demonstrated lower 2-h resting pain scores than the control group (41 patients) [MD = −13.84, 95% CI (−19.78, −7.89), *p* < 0.001]. Sequential exclusion of individual studies showed that some results shifted in direction compared with the subgroup analysis, suggesting that the findings were unstable. Details are provided in Figure 3A, Table 3, and Supplementary Table S2.

#### 3.3.3 Postoperative 4-h resting pain score

Four studies (22, 26, 28, 30) assessed postoperative 4-h resting pain scores, including 276 patients (138 in the TAPB group and 138 in the control group). A random-effects model was used. The difference between groups was not statistically significant [MD = −14.39, 95% CI (−31.71, 2.93), *p* = 0.100], and substantial heterogeneity was observed (I^2^ = 99%). Sensitivity and subgroup analyses did not identify any study or subgroup that decisively contributed to heterogeneity. Sequential exclusion of individual studies did not change the direction of the results, indicating stable findings. Details are shown in Figure 3B, Table 3, and Supplementary Table S2.

#### 3.3.4 Postoperative 6-h resting pain score

Nine studies (21, 23–30) evaluated postoperative 6-h resting pain scores, involving 543 patients (273 in the TAPB group and 270 in the control group). A random-effects model was applied. The TAPB group demonstrated significantly lower 6-h resting pain scores compared with the control group [MD = −11.09, 95% CI (−18.35, −3.84), *p* = 0.003]. Heterogeneity among studies was high (*I^2^* = 97%). Sensitivity and subgroup analyses were conducted. Subgroup analysis indicated that the timing of TAPB was an important contributor to heterogeneity. In the subgroup receiving immediate postoperative TAPB (23, 26, 28), heterogeneity was low (*I^2^* = 44%), and the TAPB group (85 patients) showed significantly lower pain scores than the control group (82 patients) [MD = −9.42, 95% CI (−12.05, −6.79), *p* < 0.001]. Sequential exclusion of individual studies did not change the direction of the effect, indicating stable results. Details are provided in Figures 3B,C, Table 3, and Supplementary Table S2.

#### 3.3.5 Postoperative 12-h resting pain score

Nine studies (21, 23–30) assessed postoperative 12-h resting pain scores, including 543 patients (273 in the TAPB group and 270 in the control group). A random-effects model was used. The TAPB group demonstrated significantly lower pain scores at 12-h postoperatively than the control group [MD = −8.82, 95% CI (−13.81, −3.82), *p* < 0.001]. Heterogeneity was substantial across studies (*I^2^* = 95%). Sensitivity and subgroup analyses did not identify any study or subgroup that had a decisive impact on heterogeneity. Sequential exclusion of individual studies did not alter the direction of the effect, suggesting stable results. Details are shown in Figure 3D, Table 3, and Supplementary Table S2.

#### 3.3.6 Postoperative 24-h resting pain score

Ten studies (21–30) compared postoperative 24-h resting pain scores, including a total of 639 patients, with 321 in the TAPB group and 318 in the control group. Meta-analysis was conducted using a random-effects model. The overall results indicated that the TAPB group had lower 24-h resting pain scores than the control group [MD = −6.9, 95% CI (−12.83, −0.97), *p* = 0.020]. Heterogeneity across studies was substantial (*I^2^* = 97%). Sensitivity and subgroup analyses were performed to explore potential sources of heterogeneity. Subgroup analysis showed that regional differences were an important influencing factor. In the non-Chinese subgroup (21–23, 28), heterogeneity was low (*I^2^* = 45%). Compared with the control group (111 patients), the TAPB group (113 patients) had significantly lower 24-h resting pain scores [MD = −5.53, 95% CI (−8.94, −2.11), *p* = 0.002]. Sequential exclusion of individual studies revealed that some statistical results were opposite in direction to those observed in the subgroup analysis, suggesting unstable findings. Details are presented in Figure 3E, Table 3, and Supplementary Table S2.

#### 3.3.7 Postoperative 2-h active pain score

**Figure 4:**
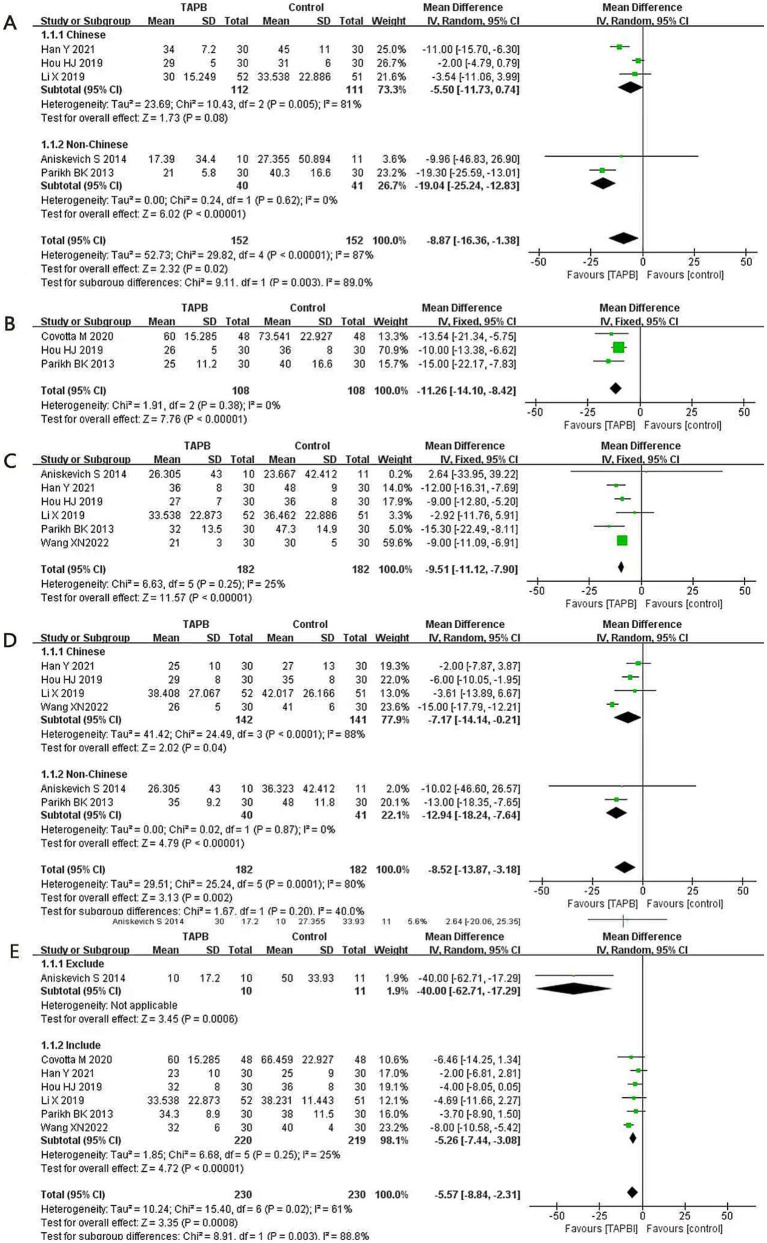
Meta-analysis of postoperative activity pain scores. **(A)** Postoperative 2-h active pain score; **(B)** postoperative 4-h active pain score; **(C)** postoperative 6-h active pain score; **(D)** postoperative 12-h active pain score; **(E)** postoperative 24-h active pain score.

Five studies (21, 24, 26–28) compared postoperative 2-h active pain scores, involving 304 patients, with 152 in the TAPB group and 152 in the control group. A random-effects model was applied. Overall, the TAPB group exhibited lower 2-h active pain scores than the control group [MD = −8.87, 95% CI (−16.36, −1.38), *p* = 0.020]. Heterogeneity across studies was high (*I^2^* = 87%). Sensitivity and subgroup analyses were conducted, and subgroup analysis indicated that regional differences were a major contributor to heterogeneity. In the non-Chinese subgroup (21, 28), heterogeneity was absent (*I^2^* = 0%), and the TAPB group (40 patients) had significantly lower 2-h active pain scores than the control group (41 patients) [MD = −19.04, 95% CI (−25.24, −12.83), *p* < 0.001]. Sequential exclusion of individual studies showed that several results shifted direction relative to the subgroup analysis, indicating unstable findings. See Figure 4A, Table 3, and Supplementary Table S2 for details.

#### 3.3.8 Postoperative 4-h active pain score

Three studies (22, 26, 28) assessed postoperative 4-h active pain scores, involving 216 patients, with 108 in the TAPB group and 108 in the control group. A fixed-effects model was used due to minimal heterogeneity (*I^2^* = 0%). The results demonstrated that the TAPB group had significantly lower 4-h active pain scores than the control group [MD = −11.26, 95% CI (−14.10, −8.42), *p* < 0.001]. Sequential exclusion of individual studies did not alter the direction of the effect, indicating stable findings. See Figure 4B, Table 3, and Supplementary Table S2 for details.

#### 3.3.9 Postoperative 6-h active pain score

Six studies (21, 24, 26–29) compared postoperative 6-h active pain scores, including 364 patients, with 182 in the TAPB group and 182 in the control group. A fixed-effects model was used due to low heterogeneity (*I^2^* = 25%). The results indicated that the TAPB group had significantly lower 6-h active pain scores than the control group [MD = −9.51, 95% CI (−11.12, −7.90), *p* < 0.001]. Sequential exclusion of individual studies revealed no change in the direction of the results, suggesting stable findings. See Figures 4B,C, Table 3, and Supplementary Table S2 for details.

#### 3.3.10 Postoperative 12-h active pain score

Six studies (21, 24, 26–29) evaluated postoperative 12-h active pain scores, involving 364 patients, with 182 in the TAPB group and 182 in the control group. Meta-analysis was conducted using a random-effects model. The TAPB group demonstrated significantly lower 12-h active pain scores than the control group [MD = −8.52, 95% CI (−13.87, −3.18), *p* = 0.002]. Heterogeneity across studies was substantial (*I^2^* = 80%). Sensitivity and subgroup analyses were used to explore potential sources of heterogeneity. Subgroup analysis indicated that regional differences were an important source of heterogeneity. In the non-Chinese subgroup (21, 28), heterogeneity was absent (I^2^ = 0%), and the TAPB group (40 patients) had significantly lower 12-h active pain scores than the control group (41 patients) [MD = −12.94, 95% CI (−18.24, −7.64), *p* < 0.001]. Sequential exclusion of individual studies did not change the direction of the findings, indicating stable results. Details are presented in Figure 4D, Table 3, and Supplementary Table S2.

#### 3.3.11 Postoperative 24-hour active pain score

Seven studies included in the initial literature (21, 22, 24, 26–29) compared postoperative 24-h active pain scores, involving a total of 460 patients, with 230 in the TAPB group and 230 in the control group. Meta-analysis was conducted using a random-effects model. The overall results indicated that postoperative 24-h activity pain scores were lower in the TAPB group than in the control group [MD = −5.57, 95% CI (−8.84, −2.31), *p* < 0.001], with substantial heterogeneity across studies (*I^2^* = 61%). Sensitivity analysis and subgroup analysis were performed to explore the sources of heterogeneity. Sensitivity analysis showed that the study by Aniskevich et al. (21) was a major contributor to the heterogeneity. After excluding this study, heterogeneity was reduced (*I^2^* = 25%), and compared with the control group (219 patients), patients in the TAPB group (220 patients) exhibited significantly lower postoperative 24-h activity pain scores [MD = −5.26, 95% CI (−7.44, −3.08), *p* < 0.001]. Exclusion of individual studies did not alter the direction of the statistical results, indicating stable findings. Details are shown in Figure 4E, Table 3, and Supplementary Table S2.

#### 3.3.12 Time to first postoperative analgesia

**Figure 5:**
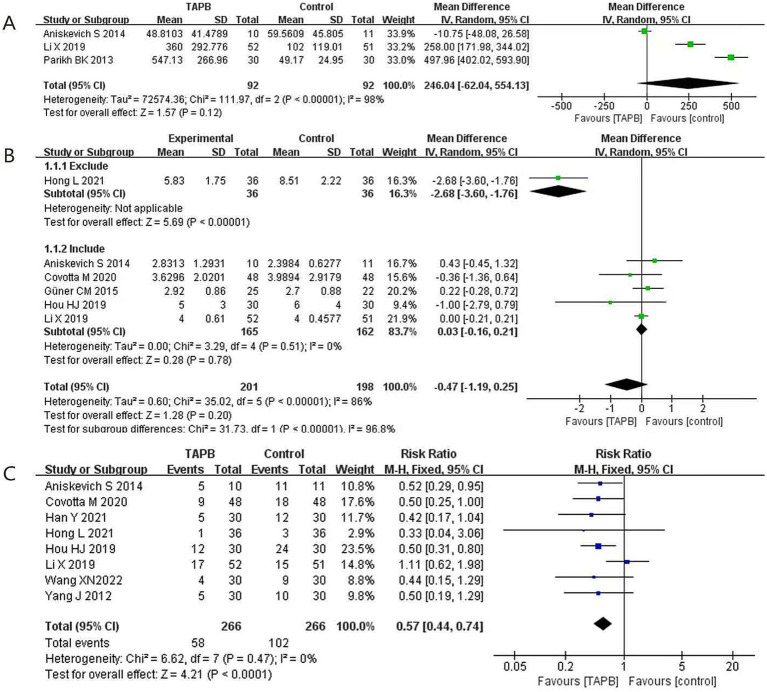
Meta-analysis of postoperative time to first analgesia, duration of hospitalization, and incidence of gastrointestinal adverse reactions. **(A)** Time to first postoperative analgesia; **(B)** duration of postoperative hospitalization; **(C)** incidence of postoperative gastrointestinal adverse reactions.

Three studies in the included literature (21, 27, 28) compared the time to first postoperative analgesia, involving a total of 184 patients, with 92 in the TAPB group and 92 in the control group. A random-effects model was applied for meta-analysis, and the results showed no statistically significant difference between the two groups [MD = 246.04, 95% CI (−62.04, 554.13), *p* = 0.120], with considerable heterogeneity across studies (*I^2^* = 98%). Sensitivity analysis and subgroup analysis were conducted to identify potential sources of heterogeneity but did not reveal any single study or subgroup with a decisive impact. Exclusion of individual studies resulted in partial inconsistency in the direction of statistical outcomes compared with subgroup analyses, indicating instability. Details are presented in Figure 5A, Table 3, and Supplementary Table S2.

#### 3.3.13 Duration of postoperative hospitalization

Six studies in the initial literature (21–23, 25–27) compared the duration of postoperative hospitalization, including 399 patients, with 201 in the TAPB group and 198 in the control group. Meta-analysis using a random-effects model showed no statistically significant difference between groups [MD = −0.47, 95% CI (−1.19, 0.25), *p* = 0.200], with high heterogeneity (*I^2^* = 86%). Sensitivity analysis identified the study by Hong L (27) as a major contributor to heterogeneity. After excluding this study, heterogeneity decreased markedly (*I^2^* = 0%), and no statistically significant difference in postoperative hospital stay was observed between the TAPB group (165 patients) and the control group (162 patients) [MD = 0.03, 95% CI (−0.16, 0.21), *p* = 0.780]. Exclusion of individual studies did not change the direction of the statistical outcomes, indicating robust findings. Details are shown in Figure 5B, Table 3, and Supplementary Table S2.

#### 3.3.14 Incidence of postoperative gastrointestinal adverse reactions

Eight studies included in the literature (21, 22, 24–27, 29, 30) evaluated the incidence of postoperative gastrointestinal adverse reactions, defined as postoperative nausea and vomiting, among 532 patients, with 266 in the TAPB group and 266 in the control group. Meta-analysis using a fixed-effects model demonstrated minimal heterogeneity (*I^2^* = 0%). The results indicated that the TAPB group had a lower incidence of postoperative gastrointestinal adverse reactions compared with the control group [RR = 0.57, 95% CI (0.44, 0.74), *p* < 0.001]. Exclusion of individual studies did not affect the direction of the results, indicating stability. Details are provided in Figure 5B,C, Table 3, and Supplementary Table S2.

#### 3.3.15 Meta-analysis results and evidence levels

The quality of evidence for the included outcome indicators, assessed using the GRADEpro system, varied across studies: one indicator was rated as high quality, five as moderate quality, three as low quality, and five as very low quality. Details are provided in Table 3 and the Annex.

### 3.4 Publication Bias

**Figure 6:**
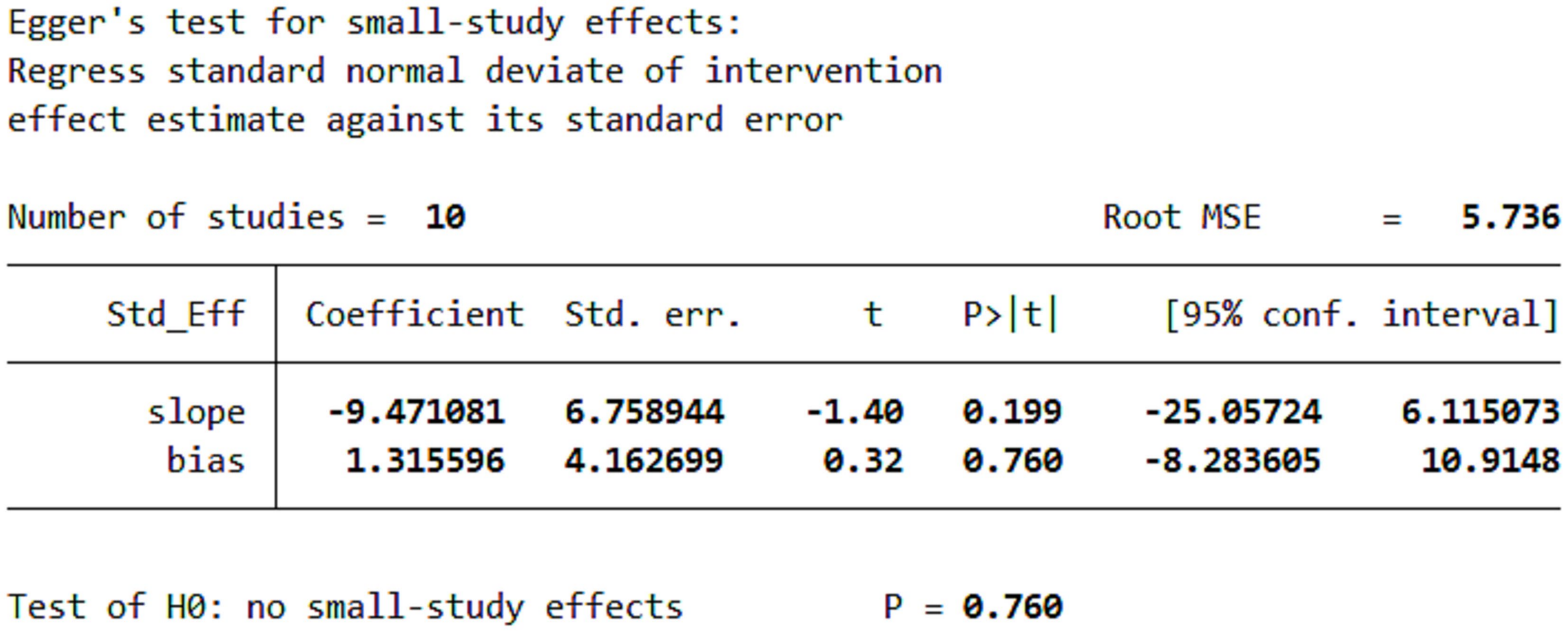
Egger’s test of postoperative 24-h resting pain score.

Among the outcome indicators, only the 24-h resting pain score was included in ten or more studies. Publication bias for this outcome was assessed using Egger’s test, and no statistically significant evidence of bias was detected (*p* > 0.05). These findings suggest that the result for this outcome is reliable and that the risk of publication bias is low. Details are provided in Figure 6.

### 3.5 Trial sequential analysis

**Figure 7:**
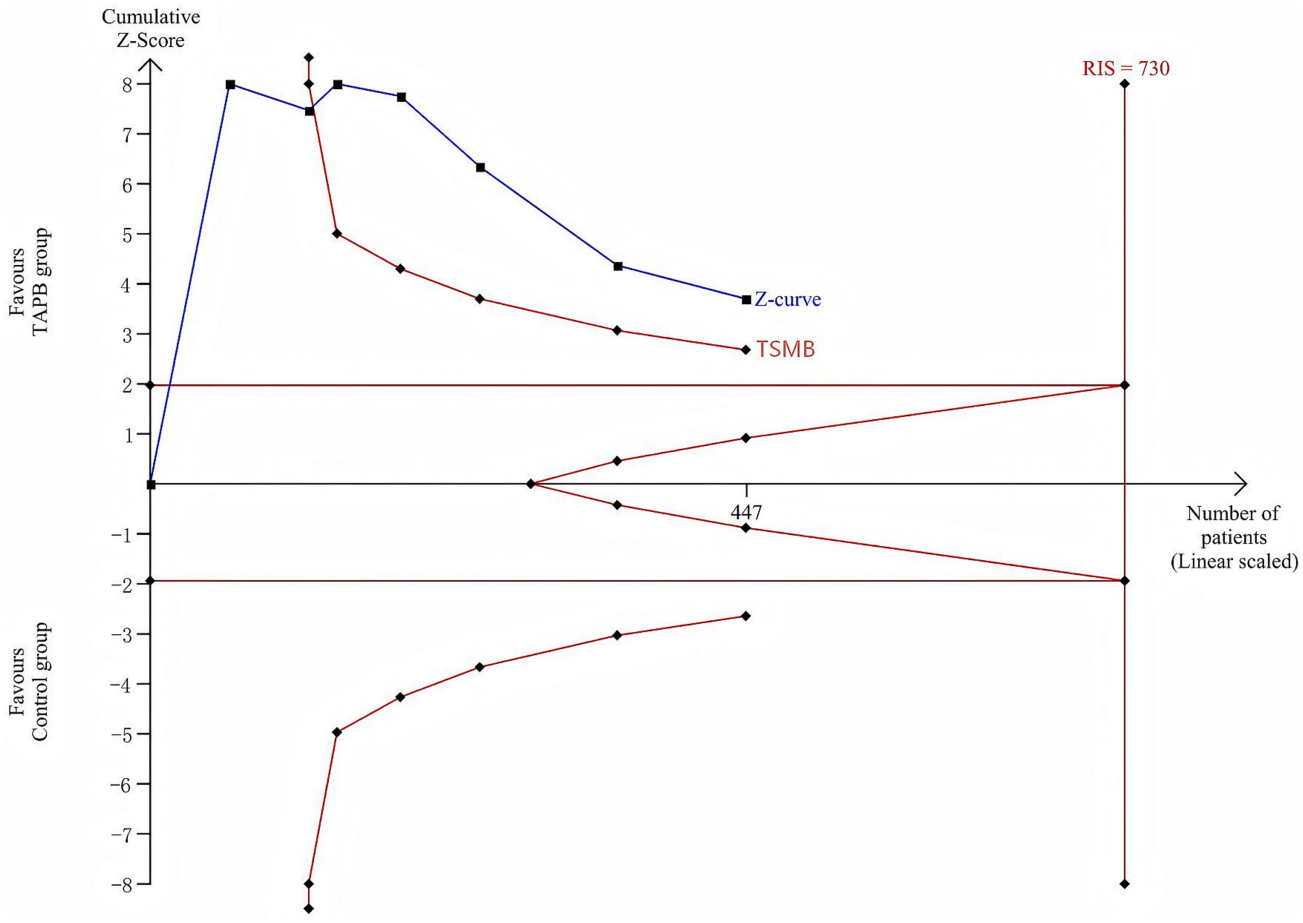
TSA of the effect of TAPB on reducing 24-h postoperative intravenous morphine equivalents.

TSA showed that the cumulative *Z*-curve for TAPB in reducing 24-h postoperative intravenous morphine equivalents crossed both the conventional boundary (*Z* = 1.96) and the trial sequential monitoring boundary (TSMB). The final included sample size (*n* = 447) remained below the required information size (RIS, *n* = 730). However, as the RIS was not reached and heterogeneity was high, the results should be interpreted with caution. Therefore, the cumulative meta-analysis provides supportive but not definitive evidence. Details are presented in Figure 7.

## 4 Discussion

The present systematic review examined whether TAPB could reduce early postoperative pain after nephrectomy. A total of 10 randomised controlled trials including 639 patients were analysed. The findings indicated that TAPB reduced postoperative 24-h intravenous morphine equivalent consumption; reduced resting pain scores at 6, 12, and 24 h postoperatively; reduced activity pain scores at 2, 4, 6, 12, and 24 h postoperatively; and decreased the incidence of postoperative gastrointestinal adverse reactions. TAPB showed no significant effect on the time to first postoperative analgesia or the length of hospital stay.

This systematic review demonstrated that the TAPB group had lower 24-h postoperative opioid consumption after nephrectomy and reduced resting pain scores at 6, 12, and 24 h and activity pain scores at multiple postoperative time points. Previous findings have shown that TAPB effectively decreases intraoperative and postoperative pain in patients undergoing abdominal surgery (32). Possible explanations include the following: (1) TAPB involves the injection of local anesthetics into the fascial plane between the internal oblique and transversus abdominis muscles, thereby blocking the afferent pathways of injurious stimuli from the T6–L1 sensory nerves that innervate the anterior abdominal wall skin, musculature, and part of the peritoneum, as well as the motor nerves of the anterior abdominal wall. This also covers the spinal segments (T10–L1) responsible for renal nociceptive transmission, resulting in reduced nociception and decreased intraoperative and postoperative opioid requirements (33, 34); (2) as part of a multimodal analgesia strategy, TAPB can be combined with other non-opioid medications and physical therapy to provide comprehensive pain control, thereby reducing reliance on a single analgesic modality; (3) TAPB may attenuate the perioperative stress response, effectively alleviate acute postoperative pain, maintain haemodynamic stability, reduce cardiovascular burden, and reduce postoperative ileus (35, 36).

The high heterogeneity for 24-h postoperative intravenous morphine equivalents (*I^2^* = 98%) may be due to differences in local anesthetic type, concentration, and dosage, variations in data reporting or conversion (e.g., median/IQR or range to mean ± SD (21, 27, 30), opioid equivalents from other analgesics (26, 28), or data extracted via GetData Graph Digitizer (23)), as well as differences in TAPB procedural approaches across studies.

This systematic review showed no statistically significant difference in the time to first postoperative analgesia between the two groups. Possible explanations include: (1) only three studies (21, 27, 28) reported this outcome, resulting in a very small sample size; (2) the quality of evidence for this outcome was rated as very low according to the GRADEpro system; (3) the included study by Aniskevich S (21), which exhibited a skewed distribution of the outcome data, altered the meta-analytic results when excluded; and (4) differences across studies in nerve block approach, timing of drug administration, and dosage and concentration of administered medications may have jointly contributed to the lack of statistically significant differences.

The present systematic review found no statistically significant difference in postoperative hospital stay between the two groups. The systematic reviews by Liu KY (37) and Tian C (38) demonstrated that TAPB reduced postoperative resting and activity pain scores in laparoscopic colon cancer and bariatric surgery but did not significantly shorten the length of hospital stay, which is consistent with the findings of the present review. Possible explanations include variability in patient populations, surgical types, anaesthetic methods, postoperative management strategies, and postoperative complications, which may substantially affect hospital stay and vary across studies.

This systematic review demonstrated that TAPB reduced the incidence of postoperative gastrointestinal adverse reactions following nephrectomy. The systematic review by Zeng J (39) found that TAPB reduced the incidence of postoperative gastrointestinal adverse reactions, which aligns with our findings. Possible explanations include: (1) TAPB primarily acts locally and reduces adverse reactions associated with excessive opioid use (40); (2) TAPB may reduce the incidence of nausea and vomiting by inhibiting the release of inflammatory mediators (41).

The application of TSA serves to mitigate the risk of type I error associated with repeated significance testing in meta-analyses, while also providing an estimate of the RIS needed to draw reliable conclusions, a methodology now well established in cumulative meta-analysis (42). In the present study, TSA showed that the cumulative Z-curve for TAPB in reducing 24-h postoperative intravenous morphine equivalents crossed both the conventional boundary and the TSMB. However, as the RIS was not reached and heterogeneity remained high, the results should be interpreted with caution, and further large-scale, well-designed, and more homogeneous trials are still warranted.

This study has several limitations: (1) some included studies reported unclear allocation concealment, incomplete outcome data, and heterogeneous surgical types, contributing to an increased risk of bias; (2) the number of included studies for several outcome measures was small (*n* ≤ 10), limiting the ability to perform robust statistical tests for publication bias, as small sample sizes substantially reduce statistical power; (3) the risk of bias in some included studies (21, 23–26, 28–30) was of some concern, and the certainty of evidence for several outcomes—including 24-h postoperative intravenous morphine equivalent, 4-h and 12-h postoperative resting pain scores, time to first postoperative analgesia, and duration of postoperative hospital stay—was rated as very low according to the GRADE assessment. Therefore, the findings for these outcomes are highly uncertain and should be interpreted with caution; (4) the included studies varied in the type, concentration, and dosage of anesthetic drugs used for nerve block, which may affect the generalizability of the pooled results; (5) In several studies (23, 25, 30), the specific conditions of pain assessment were not specified, and scores were imputed based on values from similar types of assessments, which may have introduced measurement bias, affecting the precision of pooled estimates for pain outcomes. Nevertheless, sensitivity analyses using the one-by-one exclusion method indicated that most meta-analysis results did not change in direction, demonstrating the robustness of the findings.

## 5 Conclusion

This systematic review revealed that TAPB might reduce postoperative opioid consumption, alleviate early postoperative active pain and resting pain scores at 6, 12, and 24 h postoperative, and decrease the incidence of postoperative gastrointestinal adverse reactions after nephrectomy compared with no nerve block or placebo nerve block. However, these findings should be further confirmed in rigorously designed, large-sample, high-quality randomised controlled trials, given the limited number and quality of the included studies and the low quality of evidence for several outcomes.

## Data Availability

The original contributions presented in the study are included in the article/Supplementary material, further inquiries can be directed to the corresponding author.
