# Supplementary material for: Effect of transversus abdominis plane block on postoperative pain after nephrectomy: a systematic review
Source: Front Med (Lausanne). 2026 Jun 3;13:1836287. doi: 10.3389/fmed.2026.1836287 (PMC13272322; doi:10.3389/fmed.2026.1836287)
Supplement: Supplementary file 1 [file Data_Sheet_1.docx]

**Tab. S1 Literature Search Strategy**

| Database | Retrieval type |
| --- | --- |
| Pubmed | ((((Transversus abdominis plane block[Title/Abstract]) OR (TAPB[Title/Abstract])) OR (TAP block[Title/Abstract])) AND (("Nephrectomy"[Mesh]) OR ((Nephrect*[Title/Abstract]) OR (Heminephrect*[Title/Abstract])))) AND (("Randomized Controlled Trial" [Publication Type]) OR ((((randomized controlled trial[Title/Abstract]) OR (Random[Title/Abstract])) OR (Placebo[Title/Abstract])) OR (Random*[Title/Abstract]))) |
| Embase | ('nephrectomy'/exp OR 'nephrectomy' OR nephrectomy:ti, ab, kw OR nephrectomies:ti, ab, kw OR heminephrectomy:ti, ab, kw OR heminephrectomies:ti, ab, kw) AND ('randomized controlled trial'/exp OR 'randomized controlled trial' OR 'randomized controlled trial':ti, ab, kw OR random:ti, ab, kw OR random*:ti, ab, kw OR placebo:ti, ab, kw) AND ('transversus abdominis plane block'/exp OR 'transversus abdominis plane block' OR 'transversus abdominis plane block':ti, ab, kw OR tapb:ti, ab, kw OR 'tap block':ti, ab, kw) |
| Cochrane | (Nephrectomy OR Nephrect* OR Heminephrect*):ti, ab, kw AND ((Transversus abdominis plane block) OR (TAPB) OR (TAP block)):ti, ab, kw AND ((Randomized controlled trial) OR (Random) OR (Random*) OR (Placebo)):ti, ab, kw |
| Web of Science | ((TS=(Nephrect* OR Heminephrect*)) AND TS=(Transversus abdominis plane block OR TAPB OR TAP block)) AND TS=(Randomized Controlled Trial OR Random OR Random* OR Placebo) |
| ClinicalTrials.gov | (Condition/disease:Nephrectomy) AND (Intervention/treatment:Transversus abdominis plane block) |
| apps.who.int/trialsearch | (Nephrectomy AND Transversus abdominis plane block) |
| CNKI | (Article abstract: Nephrectomy (precise)) OR (Article abstract: Partial nephrectomy (precise)) OR (Article abstract: Laparoscopic partial nephrectomy (precise)) OR (Article abstract: Renal resection (precise)) OR (Article abstract: Donor nephrectomy (precise)) OR (Article abstract: Radical nephrectomy (precise)) OR (Article abstract: Simple nephrectomy (precise)) AND (Article abstract: Transverse abdominal plane block (TAP) (precise)) OR (Article abstract: Transverse abdominal block (precise)) OR (Article abstract: TAP (precise)) OR (Article abstract: TAPB (exact)) OR (Article abstract: Trans-transversus plane block analgesia (exact)) OR (Article abstract: Trans-transversus plane block technique (exact)) OR (Article abstract: Trans-transversus plane block anaesthesia (exact)) AND (Article abstract: Randomised controlled trial (exact)) OR (Article abstract: Randomised controlled trial (exact)) OR (Article abstract: Randomised (exact)) OR (Article abstract: Randomised controlled (exact)) OR (Article abstract: RCT (exact)) OR (Article abstract: Randomised controlled study (exact)) |
| VIP Database | ((((((((Any field=Nephrectomy OR Any field=Partial nephrectomy) OR Any field=Laparoscopic partial nephrectomy) OR Any field=Nephrectomy) OR Any field=Donor nephrectomy) OR Any field=Radical nephrectomy) OR Any field=Simple nephrectomy) AND (((( ((Any field = Transverse Abdominal Plane Block OR Any field = Transverse Abdominal Block) OR Any field = TAP) OR Any field = TAPB) OR Any field = Transverse Abdominal Plane Block Technique) OR Any field = Transverse Abdominal Plane Block Anaesthesia) OR Any field = Transverse Abdominal Plane Block Analgesia)) AND ((( ((Any field = Randomised controlled trial OR Any field = Randomised controlled experiment) OR Any field = Randomised controlled) OR Any field = Randomised) OR Any field = Randomised controlled study) OR Any field = RCT)) |
| CBM | (('Nephrectomy'[unweighted: expanded]) OR ('Partial nephrectomy'[common field: intelligent] OR 'Laparoscopic partial nephrectomy'[common field: intelligent] OR "Nephrectomy"[common field: intelligent] OR 'Donor nephrectomy'[common field: intelligent] OR 'Radical nephrectomy' [Common field: Smart] OR 'Simple nephrectomy'[Common field: Smart] OR "Nephrectomy"[Common field: Smart])) AND ('Transversus abdominis plane block' [Common field: intelligent] OR 'transversus abdominis plane block'[Common field: intelligent] OR 'TAP'[Common field: intelligent] OR "TAPB"[Common field: intelligent] OR 'transversus abdominis plane block analgesia'[Common field: intelligent] OR 'transversus abdominis plane block technique'[Common field: intelligent] OR 'transversus abdominis plane block anaesthesia' [Common field: Smart]) AND (('Randomised controlled trial'[Unweighted: Expand]) OR ('Randomised controlled experiment' [Common field: Smart] OR 'Randomised controlled trial'[Common field: Smart] OR 'Randomised control'[Common field: Smart] OR "Random"[Common field: Smart] OR 'Randomised controlled study'[Common field: Smart] OR 'RCT'[Common field: Smart])) |

**Tab. S2 Tests for heterogeneity sources and sensitivity analysis**

| **Outcome Measure** | **Excluded individual studies** | **Major Source of Heterogeneity**  **(Yes/No)** | ***I²*(%)** | **Meta-Analysis Result** | | |
| --- | --- | --- | --- | --- | --- | --- |
|  |  |  |  | **Change in Statistical Significance** | **Effect Size**  **(95%CI), P value** | **Effect Model** |
| Intravenous morphine equivalents at 24-hour postoperatively | Aniskevich S 2014 | No | 99 | No | −16.28(−25.50, −7.05), *P* < 0.001 | Random |
|  | Covotta M 2020 | No | 97 | No | −19.13(−27.73, −10.53), *P* < 0.001 | Random |
|  | Güner CM 2015 | No | 99 | No | −16.91(−29.06, −4.77), *P* = 0.006 | Random |
|  | Hou HJ 2019 | No | 98 | No | −13.38(−21.70, −5.06), *P* = 0.002 | Random |
|  | Li X 2019 | No | 98 | No | −20.11(−29.51, −10.70), *P* < 0.001 | Random |
|  | Paeikh BK 2013 | No | 98 | No | −14.90(−24.53, −5.27), *P* = 0.002 | Random |
|  | Yang J 2012 | No | 98 | No | −16.06(−26.53, −5.60), *P* = 0.003 | Random |
| Postoperative 2-hour resting pain score | Aniskevich S 2014 | No | 99 | Yes | −10.27(−25.34, 4.81), *P* = 0.180 | Random |
|  | Han Y 2021 | No | 99 | Yes | −10.93(−27.04, 5.18), *P* = 0.180 | Random |
|  | Hou HJ 2019 | No | 98 | Yes | −12.12(−26.31, 2.08), *P* = 0.090 | Random |
|  | Li X 2019 | No | 99 | Yes | −12.46(−27.85, 2.94), *P* = 0.110 | Random |
|  | Paeikh BK 2013 | No | 99 | Yes | −9.42(−25.85, 7.01), *P* = 0.260 | Random |
|  | Yang J 2012 | No | 74 | No | −5.61(−10.86, −0.36), *P* = 0.040 | Random |
| Postoperative 4-hour resting pain score | Covotta M 2020 | No | 99 | No | −17.00(−38.68, 4.65), *P* = 0.120 | Random |
|  | Hou HJ 2019 | No | 99 | No | −18.88(−37.87, 0.11), *P* = 0.050 | Random |
|  | Paeikh BK 2013 | No | 99 | No | −14.84(−39.49, 9.81), *P* = 0.240 | Random |
|  | Yang J 2012 | No | 93 | No | −6.79(−14.59, 1.00), *P* = 0.090 | Random |
| Postoperative 6-hour resting pain score | Aniskevich S 2014 | No | 97 | No | −11.91(−19.38, −4.43), *P* = 0.002 | Random |
|  | Güner CM 2015 | No | 97 | No | −11.22(−20.77, −1.67), *P* = 0.020 | Random |
|  | Han Y 2021 | No | 97 | No | −11.46(−19.45, −3.48), *P* = 0.005 | Random |
|  | Hong L 2021 | No | 97 | No | −9.34(−16.64, −2.04), *P* = 0.010 | Random |
|  | Hou HJ 2019 | No | 97 | No | −11.45(−19.69, −3.21), *P* = 0.006 | Random |
|  | Li X 2019 | No | 97 | No | −12.12(−19.98, −4.27), *P* = 0.002 | Random |
|  | Paeikh BK 2013 | No | 97 | No | −10.69(−18.62, −2.76), *P* = 0.008 | Random |
|  | Wang XN 2022 | No | 96 | No | −12.86(−20.03, −5.69), *P* < 0.001 | Random |
|  | Yang J 2012 | No | 95 | No | −8.72(−14.67, −2.76), *P* = 0.004 | Random |
| Postoperative 12-hour resting pain score | Aniskevich S 2014 | No | 95 | No | −9.11(−14.19, −4.03), *P* < 0.001 | Random |
|  | Güner CM 2015 | No | 94 | No | −9.60(−15.40, −3.79), *P* = 0.001 | Random |
|  | Han Y 2021 | No | 95 | No | −9.93(−15.35, −4.50), *P* < 0.001 | Random |
|  | Hong L 2021 | No | 95 | No | −7.40(−12.46, −2.35), *P* = 0.004 | Random |
|  | Hou HJ 2019 | No | 95 | No | −9.47(−15.15, −3.80), P=0.001 | Random |
|  | Li X 2019 | No | 95 | No | −9.41(−14.75, −4.07), *P* < 0.001 | Random |
|  | Paeikh BK 2013 | No | 95 | No | −8.58(−14.15, −3.00), *P* = 0.003 | Random |
|  | Wang XN 2022 | No | 95 | No | −8.86(−15.08, −2.64), *P* = 0.005 | Random |
|  | Yang J 2012 | No | 88 | No | −6.81(−10.36, −3.25), *P* < 0.001 | Random |
| Postoperative 24-hour resting pain score | Aniskevich S 2014 | No | 97 | Yes | −5.88(−11.92, 0.16), *P* = 0.060 | Random |
|  | Covotta M 2020 | No | 97 | No | −7.26(−13.56, −0.96), *P* = 0.020 | Random |
|  | Güner CM 2015 | No | 97 | Yes | −7.20(−14.54, 0.13), *P* = 0.050 | Random |
|  | Han Y 2021 | No | 97 | No | −7.51(−13.94, −1.08), *P* = 0.020 | Random |
|  | Hong L 2021 | No | 97 | No | −7.44(−14.11, −0.76), *P* = 0.030 | Random |
|  | Hou HJ 2019 | No | 97 | No | −7.19(−13.78, −0.60), *P* = 0.030 | Random |
|  | Li X 2019 | No | 97 | No | −7.33(−13.76, −0.89), *P* = 0.030 | Random |
|  | Paeikh BK 2013 | No | 97 | No | −7.29(−13.78, −0.79), *P* = 0.030 | Random |
|  | Wang XN 2022 | No | 96 | No | −7.78(−14.20, −1.35), *P* = 0.020 | Random |
|  | Yang J 2012 | No | 60 | No | −3.63(−5.64, −1.62), *P* < 0.001 | Random |
| Postoperative 2-hour active pain scores | Aniskevich S 2014 | No | 90 | No | −8.84(−16.61, −1.06), *P* = 0.030 | Random |
|  | Han Y 2021 | No | 88 | Yes | −8.26(−18.51, 2.00), *P* = 0.110 | Random |
|  | Hou HJ 2019 | No | 71 | No | −11.45(−18.81, −4.08), *P* = 0.002 | Random |
|  | Li X 2019 | No | 90 | No | −10.41(−19.79, −1.06), *P* = 0.030 | Random |
|  | Paeikh BK 2013 | No | 71 | Yes | −5.59(−11.45, 0.26), *P* = 0.060 | Random |
| Postoperative 4-hour active pain scores | Covotta M 2020 | — | — | No | −10.91(−13.96, −7.85), *P* < 0.001 | Fixed |
|  | Hou HJ 2019 | — | — | No | −14.33(−19.61, −9.06), *P* < 0.001 | Fixed |
|  | Paeikh BK 2013 | — | — | No | −10.56(−13.66, −7.46), *P* < 0.001 | Fixed |
| Postoperative 6-hour active pain scores | Aniskevich S 2014 | — | — | No | −9.53(−11, 15, −7.92), *P* < 0.001 | Fixed |
|  | Han Y 2021 | — | — | No | −9.11(−10.84, −7.37), *P* < 0.001 | Fixed |
|  | Hou HJ 2019 | — | — | No | −9.62(−11.40, −7.84), *P* < 0.001 | Fixed |
|  | Li X 2019 | — | — | No | −9.74(−11.37, −8.10), *P* < 0.001 | Fixed |
|  | Paeikh BK 2013 | — | — | No | −9.21(−10.86, −7.55), *P* < 0.001 | Fixed |
|  | Wang XN 2022 | — | — | No | −10.26(−12.80, −7.73), *P* < 0.001 | Fixed |
| Postoperative 12-hour active pain scores | Aniskevich S 2014 | No | 84 | No | −8.47(−13.99, −2.96), *P* = 0.003 | Random |
|  | Han Y 2021 | No | 74 | No | −10.23(−15.45, −5.01), *P* < 0.001 | Random |
|  | Hou HJ 2019 | No | 78 | No | −9.17(−15.53, −2.81), *P* = 0.005 | Random |
|  | Li X 2019 | No | 83 | No | −9.26(−15.04, −3.47), P=0.002 | Random |
|  | Paeikh BK 2013 | No | 84 | No | −7.28(−13.98, −0.58), *P* = 0.030 | Random |
|  | Wang XN 2022 | No | 52 | No | −6.63(−11.16, −2.11), *P* = 0.004 | Random |
| Postoperative 24-hour active pain scores | Aniskevich S 2014 | Yes | 25 | No | −5, 26(−7.44, −3.08), *P* < 0.001 | Random |
|  | Covotta M 2020 | No | 67 | No | −5.53(−9.20, −1.85), *P* = 0.003 | Random |
|  | Han Y 2021 | No | 60 | No | −6.31(−9.91, −2.72), *P* < 0.001 | Random |
|  | Hou HJ 2019 | No | 65 | No | −6.07(−10.10, −2.03), *P* = 0.003 | Random |
|  | Li X 2019 | No | 67 | No | −5.77(−9.49, −2.04), *P* = 0.002 | Random |
|  | Paeikh BK 2013 | No | 66 | No | −6.02(−9.85, −2.18), *P* = 0.002 | Random |
|  | Wang XN 2022 | No | 53 | No | −4.87(−8.66, −1.09), *P* = 0.010 | Random |
| Time to first postoperative analgesia | Aniskevich S 2014 | No | 92 | Yes | 377.00(141.85, 612.15), *P* = 0.002 | Random |
|  | Li X 2019 | No | 99 | No | 241.61(−256.91, 740.12), *P* = 0.34 | Random |
|  | Paeikh BK 2013 | No | 97 | No | 120.72(−142.59, 384.03), *P* = 0.37 | Random |
| Duration of postoperative hospitalisation | Aniskevich S 2014 | No | 88 | No | −0.66(−1.50, 0.17), *P* = 0.120 | Random |
|  | Covotta M 2020 | No | 88 | No | −0.50(−1.33, 0.33), *P* = 0.240 | Random |
|  | Güner CM 2015 | No | 88 | No | −0.67(−1.71, 0.36), *P* = 0.200 | Random |
|  | Hong L 2021 | Yes | 0 | No | −0.03(−0.16, 0.21), *P* = 0.780 | Random |
|  | Hou HJ 2019 | No | 88 | No | −0.42(−1.19, 0.35), *P* = 0.290 | Random |
|  | Li X 2019 | No | 88 | No | −0.64(−1.80, 0.52), *P* = 0.280 | Random |
| Incidence of postoperative gastrointestinal adverse reactions | Aniskevich S 2014 | — | — | No | 0.58(0.44, 0.77), *P* < 0.001 | Fixed |
|  | Covotta M 2020 | — | — | No | 0.59(0.45, 0.78), *P* < 0.001 | Fixed |
|  | Han Y 2021 | — | — | No | 0.59(0.45, 0.78), *P* < 0.001 | Fixed |
|  | Hong L 2021 | — | — | No | 0.58(0.45, 0.75), *P* < 0.001 | Fixed |
|  | Hou HJ 2019 | — | — | No | 0.60(0.44, 0.81), *P* < 0.001 | Fixed |
|  | Li X 2019 | — | — | No | 0.48(0.36, 0.64), *P* < 0.001 | Fixed |
|  | Wang XN 2022 | — | — | No | 0.59(0.45, 0.76), *P* < 0.001 | Fixed |
|  | Yang J 2012 | — | — | No | 0.58(0.44, 0.76), *P* < 0.001 | Fixed |

**
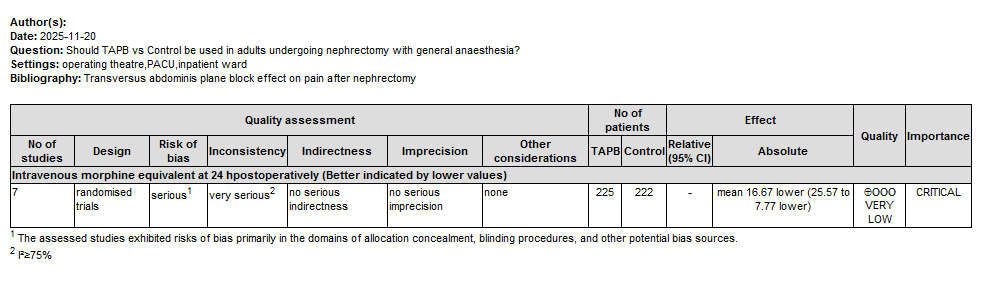
**

**Figure S1 GRADE ratings of intravenous morphine equivalents at 24 h postoperatively**

**
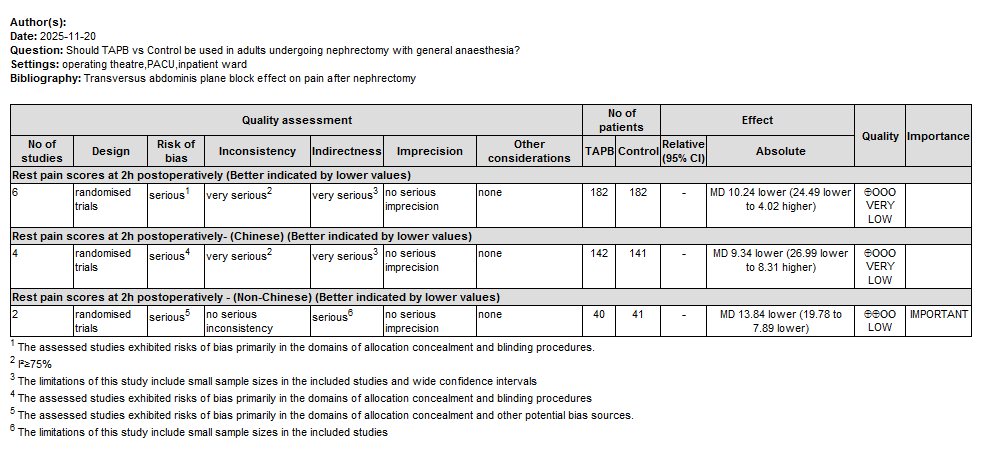
**

**Figure S2 GRADE ratings of postoperative 2 h resting state pain score**

**
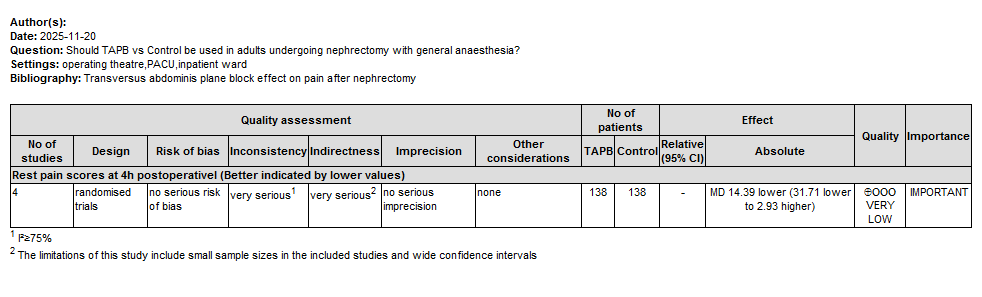
**

**Figure S3 GRADE ratings of postoperative 4 h resting state pain score**

**
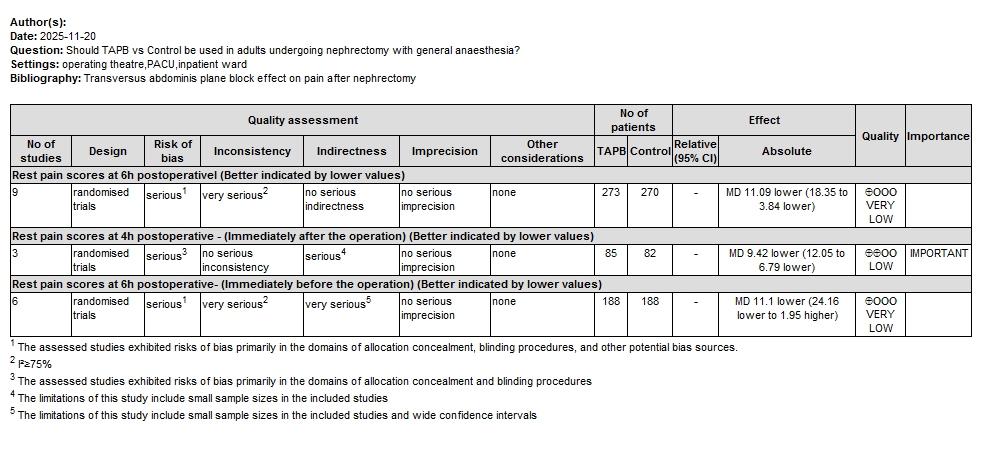
**

**Figure S4 GRADE ratings of postoperative 6 h resting state pain score**

**
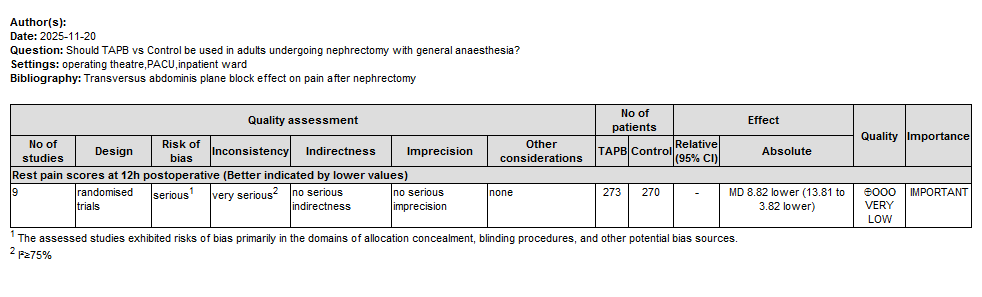
**

**Figure S5 GRADE ratings of postoperative 12 h resting state pain score**

**
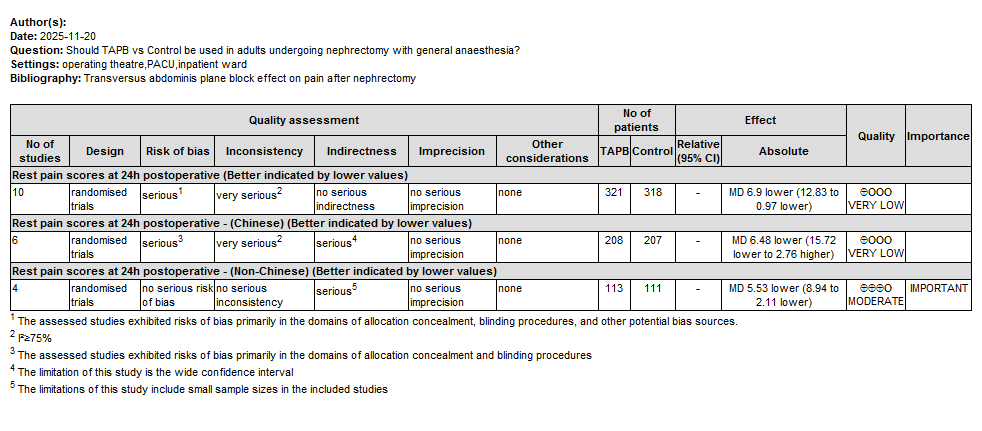
**

**Figure S6 GRADE ratings of postoperative 24 h resting state pain score**

**
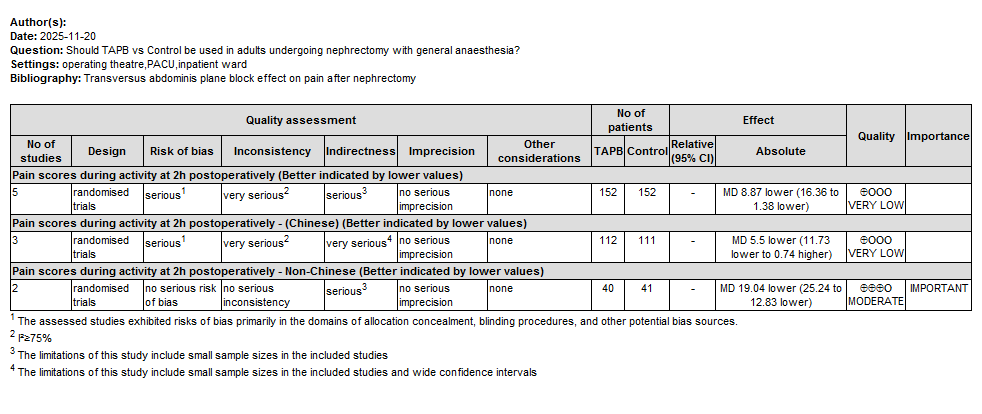
**

**Figure S7 GRADE ratings of postoperative 2 h active state pain scores**

**
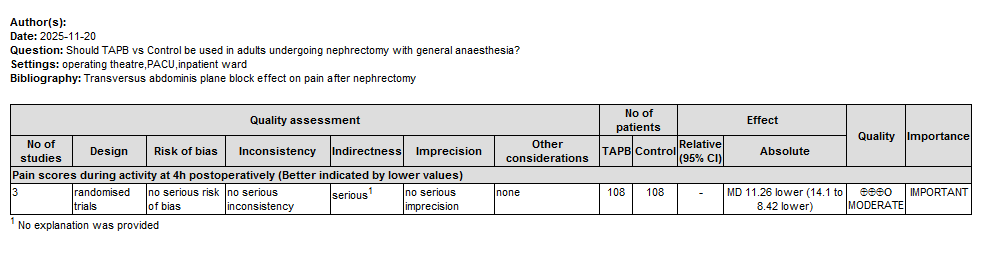
**

**Figure S8 GRADE ratings of postoperative 4 h active state pain scores**

**
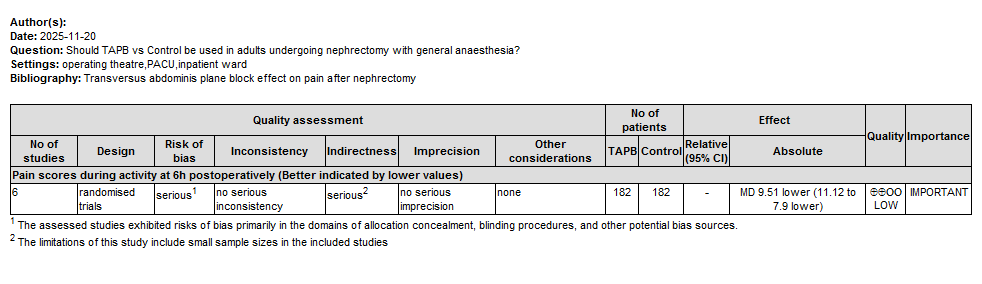
**

**Figure S9 GRADE ratings of postoperative 6 h active state pain scores**

**
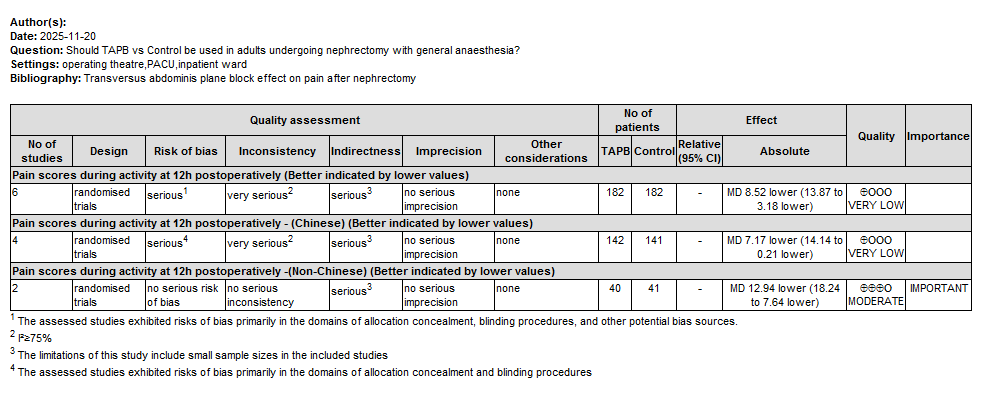
**

**Figure S10 GRADE ratings of postoperative 12 h active state pain scores**

**
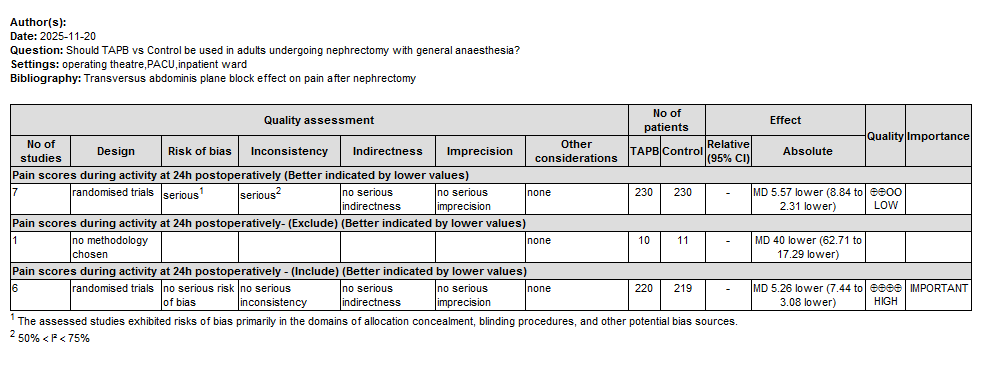
Figure S11 GRADE ratings of postoperative 24 h active state pain scores**

**
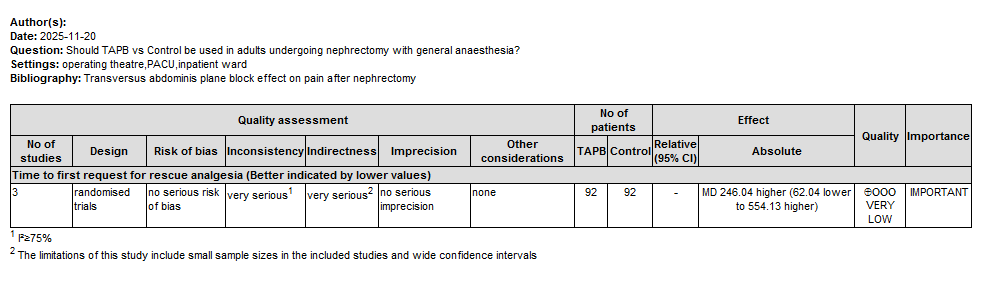
**

**Figure S12 GRADE ratings of time to first postoperative analgesia**

**
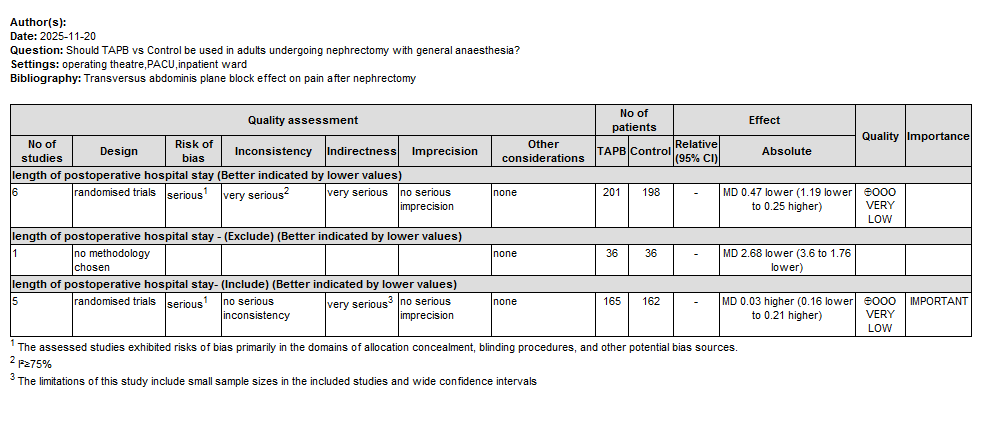
**

**Figure S13 GRADE ratings of duration of postoperative hospitalisation**

**
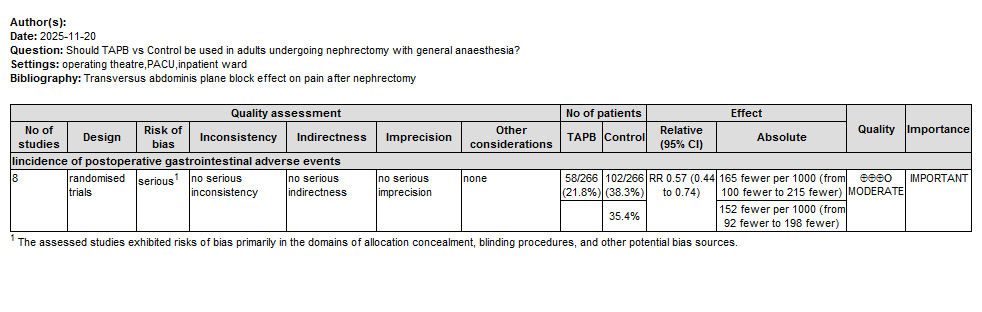
**

**Figure S14 GRADE ratings of incidence of postoperative gastrointestinal adverse reactions**
